# Regularity of Clinical Visits and Medication Adherence of Patients with Hypertension or Diabetes in Rural Yunnan Province of China

**DOI:** 10.3390/ijerph17249297

**Published:** 2020-12-12

**Authors:** Qiufeng Gao, Lanxi Peng, Wenbin Min, Jingchun Nie, Aiqin Wang, Yaojiang Shi, Haonan Shi, Dirk E. Teuwen, Hongmei Yi

**Affiliations:** 1 Center for Experimental Economics in Education, Shaanxi Normal University, Xi’an 710127, China; gqiufeng820@163.com (Q.G.); penglanxiceee@163.com (L.P.); minwenbincee@163.com (W.M.); niejingchun@yeah.net (J.N.); shiyaojiang7@gmail.com (Y.S.); 2 School of Economics and Finance, Xi’an Jiaotong University, Xi’an 710061, China; 3 Business Department Center of Red Cross Society of China, Beijing 100007, China; shihaonan2011@163.com; 4 Corporate Societal Responsibility, UCB, 1070 Brussels, Belgium; Dirk.Teuwen@ucb.com; 5 China Center for Agricultural Policy, School of Advanced Agricultural Sciences, Peking University, Beijing 100871, China; hmyi.ccap@pku.edu.cn

**Keywords:** chronic disease, rural China, self-management, hypertension, diabetes

## Abstract

Chronic diseases can be controlled through effective self-management. The purpose of this study is to explore the regularity of clinical visits and medication adherence of patients with hypertension or diabetes (PWHD), and its association with the first experience with care and individual factors in rural Southwestern China. This cross-sectional study was carried out in Yunnan province in 2018 and recruited 292 PWHD and 122 village clinics from 122 villages in 10 counties. Participants were interviewed using a structured questionnaire. Results show around 39% of hypertensive and 25% of diabetic patients neither visited physicians nor took medicine regularly during the preceding three months of the interview date. The regression results further indicated that individual characteristics of the PWHD, including patient age, health status, and economic level, as well as their first experience with care, were significantly associated with their regular healthcare behavior. In addition to providing medical services, on average each sample village clinic, with around two physicians, simultaneously managed 180 hypertensive and 45 diabetic patients. This study revealed the need for further reforms in terms of improving self-management and thus recommends an increase in the quantity and the quality of human resources in the primary healthcare realm in rural China.

## 1. Introduction

Hypertension and diabetes are not only high-incidence diseases worldwide, but also particularly serious public health problems in China. According to a study conducted by Global Burden of Disease (GBD) in 2019, the top five risks for attributable deaths worldwide were both related to hypertension and diabetes, and nearly 20% and 10% of global deaths are caused by hypertension and diabetes, respectively [1]. The prevalence in China has been rising in recent years [2,3,4]. It is estimated that the prevalence of hypertension and diabetes is around 27% and 10%, respectively, which are among the highest incidences of chronic diseases in China [5,6,7]. In 2018, cardiovascular diseases were the leading cause of mortality in China, especially for rural residents [5,8]. Moreover, as some international comparative studies have suggested, the high incidence of chronic illnesses such as hypertension and diabetes in China is a threat to global efforts to curb healthcare costs [9,10,11].

In response to these challenges, the Chinese government implemented the “National Basic Public Health Service Program” in 2009 to promote more equal access to public health services for urban and rural residents through primary medical and health institutions [12]. Counting on the support of government financial subsidies, public services including both population-based and group services (e.g., women, children, the elderly and patients with non-communicable diseases) were delivered to Chinese residents. Specifically, for patients with diabetes or hypertension (henceforth, PWHD) in rural areas, primary health institutions (i.e., township health center and village clinics) were mainly responsible for delivering regular health services, such as diagnostic and treatment services.

While effective treatments are available, roughly half of the PWHD do not self-manage correctly in rural China [13,14,15]. Self-management of chronic diseases, such as health services utilization and adherence to medicinal treatments, is mainly to assess the mastery of disease management ability of patients with chronic disease under the guidance of doctors [9,16,17]. Those patients with better self-management ability would be more likely to be in better health [17,18,19,20], and have lower social cost burden of chronic diseases [18,21]. Unfortunately, empirical findings demonstrated that a large number of PWHD in China did not use the healthcare system and failed to take medicine regularly [22,23,24]. The numbers are worse in rural areas given relatively limited medical resources and lower educational levels of residents [13,14].

In this study, we investigate the regularity of clinical visits and medication adherence of PWHD along with related factors in rural areas of Yunnan province in China. Yunnan is a multi-ethnic province where rural residents have wages below the national average (10,768 RMB vs. 14,617 RMB) [25], possibly contributing to the variation in self-management strategies. Currently, only a few studies have documented the regularity of clinical visits and medication adherence of PWHD, and how past experience with care influences their self-management in rural Yunnan [7]. Additionally, there is limited understanding of the workload of village physicians in terms of PWHD management; these physicians are the gatekeepers of chronic disease management in rural China [24].

To better understand the current self-management of PWHD in rural areas of Yunnan province in China, we (i) investigate the regularity of clinical visits and medication adherence of PWHD, (ii) document the health seeking behavior of PWHD at their first experience with care, (iii) examine factors related with the regularity of clinical visits and medication adherence, and (iv) evaluate the workload of village clinics in terms of PWHD management.

## 2. Materials and Method

### 2.1. Sampling

A cross-sectional survey was conducted in three designated prefectures (i.e., prefecture-level cities, the administrative level below the province and above the county) in Yunnan province. Yunnan province is located in the Southwestern China and its population is home to 3.4% of the total population of China [26]. The per capita GDP of Yunnan (around 5068 dollars per person) was less than the national average in 2017 (around 8777 dollars per person) [26]. The number of rural inhabitants in the three sample prefectures was around 6.5 million, amounting to a fifth of the rural population in Yunnan [27].

The sample was selected to have a certain degree of representativeness through a four-step process. Firstly, we excluded three urban counties and 11 minority counties from the three prefectures, keeping 15 Han-concentrated rural counties as our sampling frame. Secondly, we randomly selected 10 of the remaining counties. Thirdly, we used probability proportional to size (PPS) sampling, to randomly select 122 villages proportional to the number of villages in each county. Fourth, when visiting selected sample villages, we included a village clinic (henceforth, VC) that mainly serviced the sample village, and selected the leading clinician, who was the main source of medical care provision in each clinic.

In addition, we also conducted a household survey in each sample village using the following procedure. (a) We first collected the household roster from the village clinic. (b) We excluded households who lived far away (>3 km) from the nearest road and those in which no adults were in the village on the survey day. (c) We divided the eligible households into two groups by whether any of their family members were hypertension or/and diabetes patients. (d) We randomly selected six households from the households with PWHD. In principle, we randomly selected eight households within each sample village on the first day. However, we found that the unexpected bad weather made the transportation inside the village very hard, so the enumerators would be overburdened to complete the eight households. During the following survey time, we decided to only select six households within each sample village. In total, there are 116 villages that include six households for inclusion whereas 6 villages include eight households. If the number of eligible households was less than six, we randomly selected the rest from the other group. In this way, we finally gained a household sample with 744 households from 122 villages. (e) Our study mainly included PWHD from these households. The inclusion criteria were: over 18 years old; having hypertension and/or diabetes disease. Our study excluded those who were: unable to take the survey because of cognitive dysfunction or were temporarily living in the surveyed village for no more than three months. In total, 292 PWHD and directors for 122 VC were interviewed, with a response rate of 100% (Table 1).

### 2.2. Data Collection

In January 2018, we conducted a survey through in-depth interviews with each PWHD and the director of each VC for half a month. In general, it took around 2–3 h to conduct a face-to-face interview with each participant. Using a structured questionnaire, the interviews were administered by trained enumerators.

Detailed information was collected using a four-block survey. In the first block of the survey, specific questions were mainly asked about the regularity of clinical visits and medication adherence of PWHD. Specifically, we asked the participants about the level of the health facilities at which they bought the most recent medicine, whether the participant consulted with a doctor regularly and about their medicine intake in the past three months. If the participant reported he/she did consult with a doctor regularly, they were labeled as “regular clinical visits”; otherwise, they were labeled as “irregular clinical visits”. Medication adherence was measured in a similar way. The participants would receive the label “medication adherence” if they reported medicine intake regularly during the last three months. For those who either consulted with a doctor or took medicine regularly, they were labeled as “regular clinical visits or medication adherence.”

In the second part of the survey, we collected data on the health seeking behavior of the PWHD at their first experience with the healthcare system. These questions were specifically about the duration of the disease (preceding five years, five or ten years ago), source of detection (visiting physicians after clinical manifestations, physical examination and other disease visit), level of health facilities at which the participant sought medical treatment, treatment delay time (within or more than one week), and number of visits prior to diagnosis (one or more times).

In the third part of the survey, information on the demographic and socio-economic characteristics of the PWHD (i.e., individual factors) was collected. These questions assessed socio-economic factors that included: (1) age (<50, 50–59, 60–69 and 70 years and above); (2) gender (female and male); (3) educational attainment was measured by the total years of schooling the respondents had and categorized into four groups (never attended school, 1–6 years study, 7–9 years study, and >9 years study); (4) the self-health status was measured by asking “in general, would you say your health is: Excellent, good, fair, poor or very poor”. The health status of the participants was labeled as “good”, “medium” and “not good” if the answer was, respectively, “excellent or good”, “fair” and “poor or very poor”; (5) the income level of PWHD was asked by answering the question “whether your family belonged to poverty-stricken families”. The poverty line of poverty-stricken families was determined by the local Chinese government according to the income of the residents [28]. The information gathered from these questions were also used as independent variables.

Finally, we surveyed sample VC about their medical and public health services workload as well as the number of PWHD they managed. Specific questions on workload were mainly about the number of physicians in each VC, outpatients in the previous month, villages where the public services were offered, and the number of residents involved in the service. In terms of PWHD management, we asked for the number of PWHD that each VC managed, and the number of new suspected PWHD that were detected by each VC in the previous year.

### 2.3. Statistical Analysis

We used logistic regression models to analyze the associations between the regularity of clinical visits and medication adherence, and the first experience with care. We accounted for demographic and socio-economic characteristics using the three factors of predisposing, enabling and need that were derived from Andersen’s Behavioral Model of Health Services Use [29]. Logistic regression describes the above relationships through the adjusted odds ratios (ORs) and their 95% confidence intervals. All statistical analyses were performed using STATA 15.0.

### 2.4. Ethical Approval

Full ethical approval for this survey was obtained from the Peking University Institutional Review Board on 26 April 2017 (IRB00001052-17033). The board approved the verbal consent procedure. The verbal consent was obtained from local health departments and participants at the start of the survey.

## 3. Results

### 3.1. Characteristics of Sample PWHD

Characteristics of the PWHD in our sample are presented in Table 2. Nearly half of the population (55%) was female (Row 1). The average age of the respondents was around 61, and 87% of them were over 50 years old (Row 2). Nearly 43% of the respondents had never attended school; 42% did not graduate from junior high school; and 162 respondents (56%) reported poor health status (Rows 3 and 4). In addition, around one-third of the respondents (31%) came from households that were below the poverty line (Row 5).

### 3.2. Regularity of Clinical Visits and Medication Adherence

We first reported the level of health facilities that the participants selected to buy the most recent medicine (Table 3, Row 1). In short, some PWHD (27% and 15%, respectively) neither sought any treatment nor took medicine for a while. Less than one-third of the PWHD (30% and 27%, respectively) bought medicine from rural primary health centers (i.e., village clinics and township health centers), whereas 15% of hypertensive patients and 33% of diabetic patients preferred large hospitals. In addition, around 19% of hypertensive and 23% of diabetic respondents obtained their medicine from drugstores.

The self-management of chronic diseases by survey participants is also described in Table 3. The survey data show that 73% of the hypertensive patients (193) and 79% of the diabetic patients (38) did not consult with a doctor regularly (Row 2). In addition, 40% of the hypertensive patients (106) and 25% of the diabetic patients (12) did not take medication regularly in the three months prior to the study (Row 3). In general, around two-fifths (39%) of the hypertensive patients and a quarter of the diabetic patients neither visited their doctor nor took medicine regularly in the three months prior to the study (Row 4).

### 3.3. Health Care Seeking Behaviors at First Experience with Care

The participants were asked to try to recall their health care seeking behavior at their first experience with care (Table 4). In general, more than half of the PWHD (63% and 52%, respectively) had been diagnosed with hypertension or diabetes in the preceding five years (Row 1). Only 13% of hypertensive and 17% of diabetic respondents had the chronic disease for more than 10 years (Row 1). In terms of source of detection, 34% of the hypertensive patients reported that their chronic diseases were detected on visiting the clinic, whereas 41% of these patients reported that their conditions were detected through physical examinations (Row 2). Similarly, 54% of the participants diagnosed as diabetic reported that they detected their chronic illness through a visit to the clinic, whereas 27% of the diabetic participants reported that their disease was detected through physical examinations (Row 2).

We also asked questions relating to the behavior of PWHD after they were informed that they had been diagnosed with hypertension or diabetes. Most of the respondents (78% of the hypertensive and 92% of the diabetic respondents) reported they sought care from health facilities (Table 4, Row 3). Only a minority (8% of hypertensive respondents and 4% of diabetic respondents) first chose to buy directly from a drugstore. However, 14% of those diagnosed with hypertension and 4% of diabetic respondents had not sought treatment at the time of the survey (Row 3). More than half of the respondents (66% and 65%) had a doctor’s appointment within one week of being diagnosed, and most of them (92% and 93%, respectively) were diagnosed for the first time (Rows 4 and 5).

### 3.4. Factors Affecting the Regularity of Clinical Visits and Medication Adherence

Table 5 identifies factors associated with regular clinical visits or regular medicine intake in the previous three months. Among the 292 PWHD, results of the multivariate logistic regression analyses indicated that, apart from gender, the other individual variables were correlated with the regularity of medicinal intake as well (Rows 1 to 5). Specifically, compared with PWHD aged below 50, older PWHD were more likely to exhibit regularity in behavior (Row 2). Moreover, it seems that literate patients and patients with a poor health status visited physicians and took medicine more regularly (Rows 3 and 4). Economic factors, such as the income level of the household, were also significantly associated with regular health behavior patterns of PWHD (Row 5).

Additionally, the first experience with care by PWHD was also found to be associated with regular health behavior patterns (Rows 6 and 7). Compared to their counterparts, PWHD who chose to see a doctor at the first experience with care were more likely to be regular in their hospital visits and medicinal intake (Row 6). Meanwhile, the duration that had passed since the individual had been diagnosed with a chronic disease was not found to be significantly associated with the regularity with which individuals visited the hospitals or took their medicine (Row 7).

### 3.5. Workloads of Village Clinics in Terms of PWHD Management

On average, each sample VC had about two physicians (Table 6, Row 1). According to the survey data, these VCs provided both medical and public health services. Around 451 outpatients were seen at a typical VC in the month preceding the survey (Row 2). Each VC was responsible for providing a series of specified public health services (including chronic management) to about one village, which accounted for 3032 rural residents on an average (Rows 3 and 4). Specifically, around 180 hypertensive patients and 45 diabetic patients were managed by each VC (Row 5). On an average, 15 individuals were diagnosed as hypertensive whereas four individuals were diagnosed as diabetic at a typical VC in the previous year (Row 6).

## 4. Discussion

This study explored the regularity of clinical visits and medication adherence among PWHD in rural areas of Yunnan province in China. First, the data indicated that only 61% of hypertensive and 75% of diabetic patients went to see doctors or took medication regularly. Second, these patients also reported their health seeking behavior at the first experience with care and a proportion of them did not initially seek care from health facilities. Third, factors including individual characteristics and the first experience with care are significantly associated with regular healthcare behaviors of PWHD. Fourthly, in addition to providing medical services, each sample VC with around two physicians managed 180 hypertensive and 45 diabetic patients on an average.

Although low-cost medication and health services of rural primary physicians were currently available due to the health care reforms launched in 2009, PWHD self-management was still unsatisfactory. Consistent with other findings in China, our study shows that a proportion of PWHD in rural Yunnan province were not able to manage their chronic illnesses effectively [5,6,30]. Specifically, only 30% of hypertensive and 27% of diabetic respondents bought their last medicine from rural primary health institutions, whereas 27% of hypertensive and 15% of diabetic respondents did not take medicine for a while. Moreover, 39% of hypertensive and 25% of diabetic respondents in the sample neither went to see a doctor nor took medication regularly in 2018. Given that there are more than 244.5 million hypertensive and 116.4 million diabetic patients in rural China, the erratic behavior of these individuals will be a major challenge to the Chinese government’s efforts to control these diseases [7,10]. Not only in China, these challenges are also common around the world. According to estimates by the World Health Organization, around half of chronic patients in developed countries took medicine on time [31], and more than half of them did not have regular visits [32]. Some studies further found that many American patients had low levels of medicine adherence and difficulty in improving their healthy behavior [33,34], which had caused a heavy burden on the society [35]. These results highlight the urgency for more investments in primary health care, which is the first line of defense in the control of diabetes and hypertension. Improvements in primary healthcare could strengthen the ability of the state to educate the people on, and to supervise the management of, chronic diseases in rural China [36,37].

While most PWHD sought care from health facilities when they noticed they were suffering from a chronic disease, some PWHD in the sample (14% of hypertensive respondents and 4% of diabetic respondents) still had not sought any medical treatment on the day of the survey. As previous studies have suggested, improving the awareness of PWHD in rural China with regard to the self-management of their diseases is imperative [22,30,38,39]. Additionally, among the attending PWHD, some patients (34% of hypertensive and 52% of diabetic respondents) preferred higher-tier hospitals as their first source of care. This is partly due to the relative lack of trust in primary health facilities [9]. However, more than 90% of the PWHD in the sample were diagnosed on the first visit. This indicates that the capacity of primary health care in rural China has been much improved as previous findings suggested [13]. Improving the confidence of PWHD with regard to the professionalism of rural primary physicians may increase the utilization of primary health services [40].

To understand the groups of PWHD who are particularly susceptible to irregular behavior, we examined the effect of individual socio-economic characteristics of the PWHD including gender, age, educational level, health status and economic status on the proclivity to engage in irregular behavior. Our results suggested that younger and illiterate PWHD, PWHD who consider themselves to be in good health, and PWHD from low income families were more likely to exhibit irregular behavior in terms of health management. In line with some previous studies, patient age, health status and socioeconomic factors are determinants of regular healthcare service utilization [41,42,43,44,45].

Our results also emphasize the importance of recognizing the previous experiences of PWHD. Their first experiences with care were found to be associated with regular healthcare service utilization as well as regular medication intake. In line with earlier evidence, this finding suggests that seeking care from health facilities at an early stage promotes better control of hypertension and diabetes [46]. In fact, some studies further point out that not only whether PWHD received care from health providers but also how previous care was given influences the self-management of PWHD [16]. It also uncovered the benefits of educating and supervising PWHD on their initial health behaviors.

However, the results from our investigation into the workload of sample VCs show that village physicians may face challenges in improving their service quality in the management of chronic diseases. Consistent with other findings in rural China, each sample VC with two physicians not only provided medical services for hundreds of outpatients every month but also regularly delivered a series of specified public health services to one village with thousands of people [13,47]. This explained why these physicians could not effectively educate patients and supervise their behavior regarding the management of the chronic illnesses. Lack of human resources in the primary health system is one possible reason for the poor self-management of PWHD [48,49].

There are several limitations to this study. First, this was a cross-sectional study and was thus unable to identify causal relationships. Our results are merely correlation. Second, we only recruited participants with hypertension or diabetes from rural villages in three designated prefectures of Yunnan province, which limits the generalizability of the findings to this population. Thirdly, there may be selection bias and reporting bias. We only surveyed the population that had already been diagnosed with diabetes or hypertension. We were not able to include the undiagnosed PWHD in the survey. The self-reported data also cause social desirability bias and recall bias.

## 5. Conclusions

In conclusion, we found that around 39% of hypertensive patients and 25% of diabetic patients did not visit physicians or take medication regularly in the rural areas of Yunnan province. Individual characteristics of the PWHD (i.e., age, health status, and economic factors) and the first experience with care were found to be significantly associated with regular intake of medicine as well as regular visits to the hospital. Our findings suggested the urgency of improving the ability of PWHD to manage their chronic illnesses and taking adequate steps to address the shortage of primary health providers in rural China. To address the problem of poor self-management, standardized education programs should be launched to enhance public knowledge on health measures. In addition, adequate protocols for the supervision of PWHD need to be developed. These measures stress the importance of increasing the resources that the Chinese government invests in public health.

## Figures and Tables

**Table 1:** Sample size of patients with hypertension or diabetes.

Number of Diseases	Total	Hypertension	Diabetes
One disease	272	244	28
Two diseases	20	20	20
Total	292	264	48

Note: In the study, 20 respondents have both hypertension and diabetes diseases.

**Table 2:** Demographic and socio-economic characteristics of the participants.

Variable	Total *n*1 = 292	Hypertension *n*2 = 264	Diabetes *n*3 = 48
	Number (%)	Number (%)	Number (%)
1. Gender			
Male	132 (45)	120 (45)	22 (46)
Female	160 (55)	144 (55)	26 (54)
2. Age			
<50	39 (13)	33 (12)	6 (13)
50–59	77 (27)	68 (26)	15 (31)
60–69	103 (35)	94 (36)	17 (35)
≥70	73 (25)	69 (26)	10 (21)
3. Education level			
Never attended school	125 (43)	111 (42)	24 (50)
1–6 years study	123 (42)	113 (43)	16 (33)
7–9 years study	31 (11)	27 (10)	8 (17)
>9 years study	13 (4)	13 (5)	0 (0)
4. Self-reported health status			
Not good	162 (56)	144 (55)	35 (73)
Medium	86 (29)	78 (29)	10 (21)
Good	44 (15)	42 (16)	3 (6)
5. Poverty-stricken family			
Yes	90 (31)	81 (31)	18 (38)
No	202 (69)	183 (69)	30 (62)

**Table 3:** Clinical visit regularity and medication adherence.

Variable	Hypertension *n*1 = 264	Diabetes *n*2 = 48
	Number (%)	Number (%)
1. Level of health facilities selected to buy medicine last time		
No treatment or did not take medicine for a while	71 (27)	7 (15)
Village clinic	58 (22)	6 (12)
Township health center	22 (8)	7 (15)
County and upper level of hospitals	39 (15)	16 (33)
Drugstore	50 (19)	11 (23)
Others ^a^	24 (9)	1 (2)
2. Clinical visits regularly in the last three months		
Yes	71 (27)	10 (21)
No	193 (73)	38 (79)
3. Medication adherence in the last three months		
Yes	158 (60)	36 (75)
No	106 (40)	12 (25)
4. Clinical visits or medication adherence regularly in the last three months		
Yes	161 (61)	36 (75)
No	103 (39)	12 (25)

Note: ^a^ Some participants bought medicine from private clinics or bought traditional Chinese medicine.

**Table 4:** Health care seeking behavior at first experience with care.

Variable	Hypertension Sample *n*1 = 264	Diabetes Sample *n*2 = 48
	Number (%)	Number (%)
1. The duration of the chronic disease ^a^		
Preceding five years	167 (63)	25 (52)
Five years ago	64 (24)	15 (31)
Ten years ago	33 (13)	8 (17)
2. Source of detection		
Visiting physicians after clinical manifestations	91 (34)	26 (54)
Physical examination	108 (41)	13 (27)
Other disease visit	65 (25)	9 (19)
3. Level of health facilities selected to seek medical treatment at first visit		
No treatment	38 (14)	2 (4)
Take medicine at the drugstore	21 (8)	2 (4)
Clinical visits ^b^	205 (78)	44 (92)
Village clinic	63 (24)	6 (13)
Township health center	39 (15)	13 (27)
County and upper level of hospitals	90 (34)	25 (52)
Others ^c^	13 (5)	0 (0)
4. Treatment delay at first visit ^d^		
Within 1 week	150 (66)	31 (65)
>1 week	76 (34)	17 (35)
5. Number of health provider visits before first diagnosis ^d^		
1 time	207 (92)	43 (93)
>1 time	19 (8)	3 (7)

Note: ^a^ If the respondent simultaneously had hypertension and diabetes, we calculated the duration of the chronic disease based on the minimum duration. ^b^ For the respondents who selected clinical visits, we also reported the particular level of facilities they went to. ^c^ Some participants went to private clinics or sought treatment with traditional Chinese medicine. ^d^ Since 38 hypertensive and 2 diabetic respondents did not seek any treatment, we did not gather information on treatment delay and number of health provider visits from these respondents.

**Table 5:** Results of the multivariate logistic regression analyses examining factors associated with regular clinical visits and medication adherence.

Variable	Clinical Visits or Medication Adherence in the Last Three Months			
	Coef	Adjusted OR	95% CI	*p* Value
1. Gender				
Male (ref)		1.00		
Female	0.33	1.39	0.74–2.61	0.299
2. Age				
<50 (ref)		1.00		
50–59	1.10	3.02	1.20–7.59	0.019
60–69	1.11	3.03	1.22–7.52	0.017
≥70	1.03	2.79	1.09–7.16	0.032
3. Education level				
Never attended school (ref)		1.00		
1–6 years study	0.60	1.83	0.95–3.53	0.071
7–9 years study	0.75	2.12	0.73–6.17	0.169
>9 years study	1.41	4.09	0.87–19.28	0.075
4. Self-reported health status				
Not good (ref)		1.00		
Medium	−0.72	0.49	0.26–0.90	0.022
Good	−0.69	0.50	0.22–1.14	0.099
5. Poverty-stricken family				
Yes (ref)		1.00		
No	0.68	1.97	1.08–3.62	0.028
6. Clinical visits at first experience with care				
Yes (ref)		1.00		
No	−1.95	0.14	0.07–2.90	<0.001
7. The duration of the chronic disease				
Preceding five years (ref)		1.00		
Five years ago	0.45	1.58	0.81–3.08	0.184
Ten years ago	0.24	1.28	0.52–3.12	0.593

Note: ref: the reference group; Coef: regression coefficient; OR: odds ratio; CI: confidence interval.

**Table 6:** Hypertension and diabetes management among sample village clinics.

Variable	Mean	SD
1. Number of physicians per VC	2.03	0.98
2. Number of outpatients per VC in the previous month	450.63	650.91
3. Number of villages each VC provided public health services for	1.03	0.46
4. Population per VC	3032.15	1602.56
5. Number of patients managed per VC ^a^		
Hypertension	179.70	125.99
Diabetes	44.51	39.14
6. Number of new suspected patients that were diagnosed in the last year		
Hypertension	14.58	19.65
Diabetes	3.50	5.25

^a^ All of the sample village clinics reported as responsible for providing public health services. Only one of these clinics was reported as not responsible for diabetes.

## Data Availability

All replication files are available from the Harvard Dataverse: https://doi.org/10.7910/DVN/RGNCFJ, DRAFT VERSION, UNF:6:P8ZeTK5N6Zzlzuu4pTAWYw== [fileUNF].
